# Diseases of the Rectum

**Published:** 1838-04-01

**Authors:** 


### Medico-Chirurgical Review,

### No. LVI.

[No. 16 of a Decennial Series.]

JANUARY 1, 1838, to APRIL 1, 1838.

### DISEASES OF THE RECTUM.

I. A Treatise on the Malformations, Injuries, and Diseases of the Rectum and Anus. Illustrated with Plates. By *George Bushe*, M. D., formerly Professor of Anatomy and Physiology, &c. 8vo., pp. 299. New York, 1837.

II. On the Diseases of the Rectum. By *James Syme*, F.R.S.E., Professor of Clinical Surgery in the University of Edinburgh. 8vo., pp. 138. Edinburgh, 1838.

In our last number, we devoted many pages to the Authors before us and to the subject on which they treat.—Authors who deserve attention—a subject of no small importance. It would be idle dwelling again on the imperfect state of general knowledge with respect to diseases of the rectum. Nor does it appear less idle to re-enter on the strong considerations, which have induced us to devote several articles to the diffusion of as much information, as the latest works convey.

In our last notice, we arrived at the subject of ulceration of the rectum. It occupies the twelfth chapter of Dr. Bushe's work, and is not treated of at all in Mr. Syme's.

The thirteenth chapter of Dr. Bushe's work, alludes to venereal ulcerations of the rectum. It only alludes to them, and in a page and a half they are dismissed. The observations are too brief to merit attention. Mr. Syme makes no reference whatever to these ulcers. We shall commence with

### 1. Hæmorrhoidal Affections.

The fourteenth chapter of Dr. Bushe's book is devoted to "Affections called Hæmorrhoidal." The second chapter of Mr. Syme's is on "Hæmorrhoids." We shall compare the two authors, and severally introduce them to our readers. We prefer Dr. Bushe's plan and arrangement to those of Mr. Syme, and shall adopt them.

*Congestion of the Hæmorrhoidal Vessels.*

From the operation of various causes, the vessels of the rectum and anus, and more especially the veins, are liable to be preternaturally distended with blood. This congestion may subside, or give rise to hæmorrhage, or to the formation of tumors at the anus, or to inflammation, or to mucous discharge.

Dr. Bushe describes very fully the phenomena attendant on this congestion of the gut. There is generally, he says, a sense of weight and fulness in the rectum and perineum. Usually too, some of the following symptoms are present: rigors,—rigidity and occasional spasm of the extremities,—pallor,—dinginess of the skin beneath the eyes,—cold, strictured and dry skin,—weight and pain in the forehead,—vertigo,—dryness of the fauces,—white tongue,—vomiting,—temporary augmentation of the liver,—flatulence,—pain in the abdomen—constipation,—scanty and colourless urine,—increased velocity, hardness and contraction of the pulse,—precordial anxiety,—palpitation,—stricture of the epigastrium,—syncope,—hurried respiration,—a feeling of weight in the loins, hips and groins,—dull throbbing pain in the rectum, attended with a sense of increased heat, tenesmus, mucous discharge, and occasional darting sensations resembling those of electricity, itching of the anus,—and finally, painful, difficult, and frequent micturition. This state of congestion of the rectal vessels is apt to occur from time to time, and, sooner or later, at one period or another, one or more of the consequences already mentioned, and now to be more particularly dwelt on, follow.

A. *Hæmorrhage*.—This is a very prominent and important symptom and well deserves attention as a substantive affection. The bleeding, observes Dr. Bushe, usually occurs during defecation, sometimes preceding, but generally following the passage of the feces. Frequently, the loss of even a small quantity of blood relieves the feeling of weight and tension in the perineum, rectum, and lower part of the back, as well as any other disagreeable symptoms which may have existed. The amount of hæmorrhage, however, is not always in proportion to the severity of the symptoms denoting the loaded state of the hæmorrhoidal vessels. Generally it ceases after a few days, yet not unfrequently, it continues for months. In some instances it occurs but once in life; again it may return in the course of a few weeks, months, or even years. Occasionally it assumes a periodical character, returning with the season or the month.

The amount of blood lost varies; a drachm, an ounce, or even a pint may be discharged at a time, though it must be confessed that, the admixture of other fluids is apt to impose upon the inexperienced, the belief that the loss of blood is much greater than it really is.

In a note Dr. Bushe cites a few instances of the amount to which hæmorrhage, and of the far greater amount to which credulity may go. Thus, Montanus, according to the report of Schwevcher, saw a patient who had passed two pounds of blood for forty-five successive days, and finally recovered. (*Append. consilior. Montani*, p. 59. *Basil*, 1588.) Cornarius mentions the case of a gentleman who, after drinking freely of Hungarian wine, lost two pounds of blood from the nose, and six pounds on each of the four following days from the anus. Nevertheless he got well without any remedy. (*Observ. med.*, 26.) But Panarola tells a better story, for he knew a Spanish Nobleman, who, for forty years, rendered each day a pint of blood *per anum*, and at the same time enjoyed perfect health. Bozelli beats Panarola hollow. He mentions the case of a tailor, who lost as much as ten pounds of blood at a time. This man was nevertheless vigorous and of a jovial character. Bozelli diminished this flux by means of the syrup of roses. Spidler saw a potter, who after having suffered for a week with pain in the loins, was seized with violent colic, and severe vomiting. A cathartic was administered, which relieved him; but he passed from twelve to fourteen pounds of vermillion-coloured blood from the anus, in twenty-four hours, each dejection being accompanied with a slight colic pain. After many remedies were tried in vain, the hæmorrhage was arrested by a stimulating injection. (*Observ. med.* 44.) Hoffman says he saw a widow, fifty years old, of a very full habit, who in consequence of an indolent course of life and full living, was for eight years subject to hæmorrhoids, at the same time that she continued to menstruate. The uterine discharge having ceased, and being blooded but once, she was seized, towards the autumnal equinox, first with lassitude, and then with coma, for which she was bled in the foot, and took cold water in large quantities without any benefit. At the end of two days, however, a stimulating lavement was administered, when an excessive flux of blood occurred, amounting in twenty-four hours to more than twenty pounds; the consequence of which was, a cessation of the coma. Her strength gradually returned by the employment of invigorating and gently astringent remedies, together with enemata of cold water. Smetius relates the case of a man forty years of age, who passed per anum at least thirty pounds of blood in two or three days. He was cured by a tonic plaster. Twenty pounds of blood in four and twenty hours are a respectable quantity for an honest woman to get rid of.

The character of the hæmorrhage deserves a few words. Dr. Bushe remarks that the blood evacuated, is of a bright vermillion colour, and is exhaled by the extremities of the capillary vessels, as may be demonstrated by an examination of the mucous membrane when protruded, an occurrence which very often takes place in these cases. In some instances, fine streams of blood are seen to issue from dilated pores, which we are afterwards unable to detect. Besides this evidence of the source of the discharge, the symptoms which precede the flow of blood, their subsidence on the occurrence of hæmorrhage, together with the colour of the blood, plainly demonstrate the nature of the attack. However, when the bleeding has been profuse, the vessels may become so debilitated, as to allow the blood to escape from their extremities, constituting passive hæmorrhage.

Whether the blood issues from "dilated pores," may, perhaps, admit of question. It is common enough for it to issue from protruded mucous membrane in a fine continuous jet. Mr. Syme observes that the bleeding takes place when the tumors are protruded beyond the sphincter, and varies in amount from a few drops to several ounces. The blood sometimes seems to ooze from the surface, and at other times springs out in a jet, extending, if permitted, to the distance of several feet; whence it is often supposed that the patient has ruptured a blood-vessel. The quantity lost at each time of going to stool is very unequal, and varies with the condition of the patient, increasing when there is general irritation of the system or excitement of the pelvic viscera, and diminishing in circumstances of an opposite kind. For weeks or months the hæmorrhage may cease altogether, and then return more vigorously than ever; but its general tendency is to increase with the duration of the complaint.

The effects of the hæmorrhage upon the general health are a subject of great practical importance. The notion that it is salutary and should not be repressed has taken deep root in the public, some, perhaps, in the professional mind. That notion is partly founded in truth, partly in exaggeration. Without quoting cases which reading will supply to all well-informed medical men, we are tempted to extract some communicated by Dr. Bushe, because we think they put the matter on its proper footing. Dr. Bushe mentions two which proved fatal.

*Case.* 1. A gentleman between fifty and sixty, of short stature and full habit, who for many years had been subject to a profuse discharge of blood from hæmorrhoidal tumors, underwent an effectual operation for their removal, and died in seven months afterwards of apoplexy.

*Case* 2. Another gentleman, under thirty, for a few years laboured under a free hæmorrhoidal discharge, which, as it debilitated him, was checked by astringents. In a short time, however, he was attacked with pulmonary hæmorrhage, and, at the end of a year and a half, he sank, after a profuse discharge of blood.

These cannot be considered as conclusive instances of the dangers resulting from the stoppage of hæmorrhoidal bleeding. Yet, although not logically conclusive, they are practically calculated to inspire caution, and to lead the surgeon to adopt all possible means of warding off such dangerous results of operations intended for cure. Those means are very obvious. The patient should live sparingly, keep his bowels open, take ample exercise—in short he should do what will tend to prevent his making too much blood, and will carry off by the secretions and excretions the superfluous parts of what he does make. If such precautions are neglected by surgeon and patient, Dr. Bushe shews us what may, and what frequently will follow.

"If in spite of these means," (exercise, &c.), says Dr. Bushe, "the patient be threatened with congestion of any other organ, he ought to immerse his feet in warm mustard water every night, or else, sit in a hip bath, as hot as he can bear it,—to have six leeches applied to the anus every second day,—and, to take a pill consisting of one grain of calomel, half a grain of ipecacuanha, and three grains of the extract of aloes, every eight hours, until his symptoms are moderated. In this way, great danger may frequently be averted, as I have had much proof; but, to illustrate the point better, I shall give the outline of three cases.

Mr. S., on whom I operated for bleeding hæmorrhoidal tumours, lived sparingly, and took exercise for some months, by which he was restored to excellent health. He then gave up his exercise, and lived well, taking a moderate quantity of wine daily. In nine months after the operation, and two after his change of regimen, he had an apoplectic seizure, from which he recovered under antiphlogistic treatment. He then enjoyed good health, by taking five grains of the extract of aloes, two of blue mass, one of ipecacuanha, and two of ginger, in two pills every night, and applying four leeches to the anus every morning, at the same time exercising much, and living very sparingly. After a few months, he gradually sunk into his usual way of life, and had another slight apoplectic attack, which, however, soon subsided, under bleeding, purging, and revulsion on his extremities. Since his recovery, which is now more than eighteen months, he has lived altogether on vegetable food, has drunk nothing but water, and has ridden on horseback from ten to twenty miles a day, when the weather permitted. His health has been good; but, occasionally he has suffered from vertigo, which has yielded to stimulating pediluvia, and the pills above mentioned.

I removed five large hemorrhoidal tumours from Mr. L., and gave him directions, as to the course of life he ought to pursue. He did not attend to these, and consequently was attacked with spitting of blood, for which I was consulted, and recommended leeching the anus twice a week; two pills containing five grains of the extract of aloes, and five of blue mass, at bed time; a solution of the sulphate of soda, in the morning; low diet; and, after the spitting of blood ceased, regular exercise. He took altogether, twenty-four pills, three ounces of the neutral salt, and applied two dozen and a half of leeches, when he felt himself perfectly recovered, but weak. It is now fourteen months since the spitting of blood ceased, and by exercise and low living, he enjoys uninterrupted good health.

Mr. E., from whom I removed four bleeding hæmorrhoidal tumours, was attacked, two months after the operation, with spasmodic cough, and irritation of the membrane of the larynx, in consequence of which, he expectorated an immense quantity of a frothy tenaceous matter. He was an immense eater, and a man of sedentary habits. I had a great difficulty in conquering these failings, but, when I did so effectually, with the aid of warm hip bathing, leeches to the anus, and pills, consisting of the extract of aloes, calomel, ipecacuanha, and extract of belladonna, he gradually recovered." 194.

But it must not be supposed, that the hæmorrhage is generally useful, or that its arrest is generally prejudicial. Continued bleeding from the bowel occasions all the consequences of excessive bleeding from other sources. The patient loses flesh, has a waxen complexion, is listless, has a quick pulse, palpitations, dyspnœa, incapability of much exertion, either of body or mind.

Few surgeons have escaped seeing instances of this description, and few too have failed to witness the safety and advantage with which they may be frequently checked by operations. We may cite, as an instance, one detailed by Mr. Syme. He attended a lady with Dr. Donaldson, of Ayr. At an early age she had begun to suffer from hæmorrhoids, and thirty years ago had been advised by the late Mr. Benjamin Bell to have them removed. This was declined, and the disease went on increasing with all the usual symptoms, until at length the bleeding, which for seven or eight years had been very profuse, so affected the general health as to excite the serious alarm of her friends. She exhibited in an extreme degree the peculiar aspect and other symptoms of exhaustion caused by a continued drain of blood. But very soon after the removal of the hæmorrhoidal tumors, which were large and numerous, so as to encircle the aperture of the gut, she regained her strength together with a healthy look; and though three years have now elapsed since the operation was performed, she has not suffered any unpleasant symptoms from the sudden suppression of her complaint.

Though we need not apprehend fatal results whenever we arrest the hæmorrhage of piles, still, in subjects disposed to plethora, or of an age to render its occurrence likely, the prudent surgeon will enjoin after the operation those precautions which will tend to keep such injurious dispositions in abeyance.

B. *Internal Tumors.* These are so generally believed to be varices of the hæmorrhoidal veins, that many of our readers will probably be startled at the mention of any other explanation of them.

These tumors may either be within or without the anus. These have respectively received the names of "internal," and "external piles." Those situated, says Dr. Bushe, immediately within the anus, vary in number, in many instances being so numerous, as to prevent the free discharge of feces, while in other cases, they are few, even solitary. Their size is as variable as their number, differing from that of a small pea to a pullet's egg. They are generally globular, and in many instances pedunculated, particularly when large, and subject to prolapse during defecation; for under such circumstances, they swell and suffer a constriction at their bases, from the contraction of the sphincter. Generally, they are of a dark red colour, and when prolapsed, they become perfectly livid, in consequence of the obstruction created to the return of the venous blood; firstly, by the forced expirations necessary for the act of defecation, and secondly, by the constriction of the sphincter.

Mr. Syme remarks that the tumors seated within the sphincter, and capable of being forced into view by powerful expulsive efforts, possess an irregularly round form, a florid colour, a granular uneven surface, and very vascular structure, so as to bleed freely from the slightest injury. They resemble a strawberry very much in appearance, and seldom existing singly, in general constitute a more or less complete annular swelling, which, when protruded beyond the anus, seems to close the aperture of the gut completely, and is surrounded by an external ring proceeding from distention of the neighbouring loose texture, which is the seat of external hemorrhoids.

*a.* These tumors, as we have observed, are by the great majority of medical men considered as varices of the hæmorrhoidal veins. But, at various times, another opinion has been entertained. Dr. Bushe informs us:—

"I have repeatedly injected these tumours with coloured water, both from the arteries and veins, and when cut into while the fluid was projected, small jets were observed to issue from many points. I have frequently dissected them with the greatest care, and found that they were spongy, reddish, and contained both arteries and veins, the latter being most capacious, but always perfectly healthy. Their surface is villous, and generally bleeds when touched roughly, or scratched with the nail, the blood which issues being of a florid red colour. In many instances, I have been able to rub off exceedingly vascular and fragile adventitious membranes from their surface. Thus, it would seem, that they may acquire an increase of magnitude in this way." 152.

Mr. Syme observes—

"The substance of the internal hemorrhoidal tumour is so vascular and disposed to bleed, especially when forced beyond the sphincter, that it has been considered similar to the erectile tissue which composes aneurism by anastomosis and nævus. But these diseases are, with few if any exceptions, of congenital origin; while internal hemorrhoids rarely make their appearance before the age of maturity; and the vessels of the latter growth, instead of being dilated into the cellular-looking structure which composes the former, are small and arborescent. There hence does not appear to be any analogy between the two morbid structures farther than their disposition to bleed." 59.

*b.* Dr. Bushe proceeds to describe the progress of the tumors. When small they are generally attended with slight heat and itching, but as they enlarge they produce a disagreeable sense of fulness in the lower extremity of the rectum, and are prolapsed during defecation, after which they gradually shrink up, and by the action of the muscular apparatus of the anus are returned to their original situation. In some cases, however, the sphincter becomes more or less relaxed, and these tumors in descending drag along with them a portion of the adjacent mucous membrane. Indeed, so large is the protrusion, that persons thus afflicted are compelled to return it with their fingers, and many of them postpone the calls of nature, until they are about to retire for the night, in consequence of the difficulty they experience, and the time they require to reduce it, and above all, as they can only effect this in the horizontal position. In many cases, the protrusion occurs, when the patient walks, or even attempts to ride in a carriage, and thus gives rise to great uneasiness and mucous discharge. Besides the protrusion of the mucous membrane now described, that of the pouch frequently takes place from the constant nisus these tumors are apt to create.

In a few cases, when there is but one tumor, it is situated low down, and though not large, partially projects through the sphincter and gives rise to very great annoyance.

In consequence of the difficulty occasioned by defecation to the return of the venous blood, the latter accumulates in the rectum, and these tumors exhale it, perhaps in large quantities. Prior to the formation of these tumors, the mucous membrane may furnish the hæmorrhage, and after their removal it may do so again, a fact which Dr. Bushe has witnessed, and which, we may add, we have witnessed too.

c. Both Dr. Bushe and Mr. Syme insist on a leading and deceptive feature of this disorder—the protrusion of the tumors and of the mucous membrane. Mr. Syme speaks most fully upon this point, and, as it is one of great importance, we extract his observations.

"The protrusion of the swellings is a nearly constant symptom of the disease, and is troublesome merely in proportion to their size. At first the tumours pass beyond the sphincter only during the forcible and continued efforts to evacuate the bowels which attend constipation; but by-and-bye they descend more readily, and return with more difficulty, requiring to be pushed up by external pressure; and in cases of old standing, where the skin lining the anus, from being frequently put upon the stretch, remains permanently relaxed, hanging in folds round the orifice, the tendency to protrusion is so great, that the hemorrhoids descend not only on all occasions of going to stool, but also whenever the patient makes the slightest exertion, or even when he simply assumes the erect posture. The protruded part is of course painful, especially when subjected to pressure, and, by soiling the patient's clothes with the mucous and bloody discharge that issues from its surface, is a constant source of vexation. A middle aged lady, whom I saw with Dr. Begbie, had been confined for two years to the horizontal posture by hemorrhoidal swellings, which descended from the gut whenever she attempted to walk or stand. After the disease was removed she could walk for miles without any inconvenience.—A gentleman, about 50, whom I saw with Dr. Davidson, had suffered for upwards of eighteen years from a protrusion of this kind, and, holding an office in the courts of law, which frequently required him to sit for many hours in public, endured more distress than it is easy to describe or imagine. He was completely relieved by removal of the enlargement.—A man, about 40, from Dundee, was lately in the hospital here under my care on account of a hemorrhoidal protrusion, which had troubled him for more than twenty years, and latterly disabled him entirely for his occupation, which was that of a weaver. He returned home quite well.—Many other cases could be mentioned in illustration of the protrusion of the tumours constituting the prominent feature of the disease. It is such cases which generally go under the title of *Prolapsus ani*, and, being supposed to depend upon weakness of the sphincter, are palliated very imperfectly by the application of bandages to support the gut. Such means of palliation are no less unpleasant than inefficient, and in some respects, indeed, may be considered as even more irksome than the disease itself. It is therefore of the utmost importance to take a correct view of the derangement, which leads to an easy, safe, and effectual remedy." 66.

The distinctions between protruded tumors and prolapsus of the mucous membrane, or polypi, are well drawn by Dr. Bushe. The form of prolapsus, he says, with which they are likely to be confounded, is that chronic affection in which a flap of the mucous membrane, on either side, is forced down and becomes thick and rugous. However, the semilunar form of these flaps, the extent of their base, our ability to glide the folded membrane between the finger and thumb, as well as their freedom from erection and hemorrhage, are characters so opposite to those which we have described as pertaining to hemorrhoidal tumours, that a very cursory examination enables us to distinguish between them.

The slow, progressive, and indeterminate increase of every species of polypi; their incapability of erection or collapse; their large size; their pale red colour; their very soft spongy feel, when of the mucous species; their solidity, when they possess a fibrous structure, together with their freedom from inflammation and ulceration, unless when of a malignant character, or when under the influence of an irritating cause, enable us to avoid confounding hemorrhoidal tumors with them.

*d.* These tumors, even when free from congestion, and producing little immediate uneasiness, occasionally give rise in nervous individuals, to contraction of the sphincter ani and exquisite pain, which, when violent, extends to the uterus, vagina, and external organs of generation in the female, to the perineum and testicles in the male, and to the bladder and urethra in both sexes. The constant tenesmus, strangury, and dysury which it produces, wears the patient down, giving rise to sleeplessness, anxiety and fever, and in some rare cases, so excruciating is the pain, that the patient must remain perfectly tranquil, as the least motion exasperates his suffering to an intolerable degree.

Both Dr. Bushe and Mr. Syme relate cases that merit the attention of our readers, and must tend to fix important facts upon their memories. Mr. Syme indeed dwells strongly on the irritation of the urinary organs, and on the want of any regular proportion between these and other occasional symptoms and the stage of the tumors themselves.

*Case* 1. A gentleman, about 50, suffered for years from excessive pain in the region of the bladder, with frequent desire to make water. He consulted a great many physicians and surgeons of eminence, and had at length made up his mind that the disease, in accordance with the opinion of a distinguished pathologist, was tic-douloureux of the bladder, when a medical friend thought of examining his rectum, and discovered several large internal hemorrhoids, which Mr. Syme removed to the patient's great comfort.—*Syme.*

*Case* 2. In September, 1832, Mrs. ——, aged 29, consulted Dr. Bushe. She informed him, that, in 1829, she began to menstruate irregularly and scantily, but lost blood occasionally from hemorrhoidal tumors, which were very painful. In 1820, she first experienced some difficulty in micturition, attended with shooting sensations in the vulva, which towards the end of the year became very distressing; notwithstanding, she married, but so great was her suffering that she was seldom able to cohabit, and only with exquisite pain. In 1825, her husband died, and then she menstruated more profusely, yet irregularly, while the hemorrhoidal discharge diminished, the pain became far less, sometimes disappeared altogether, but occasionally returned with severity, attended with frequent and distressing calls to void urine, tenesmus, small mucous discharge, and firm contraction of the sphincter. During these attacks, the hemorrhoidal tumors were swollen, but when they began to bleed freely, the symptoms moderated. It frequently happened, however, that this did not occur, but that the uterine flux set in, which also moderated the symptoms, but not at all to the same extent. Being about to make an advantageous match, she was desirous to obtain relief if possible; therefore, she willingly submitted to an examination, which led to the detection of five or six large hemorrhoidal tumors, attended with great tenderness, not only of the anus, but also of the external organs of generation, and spasm of the sphincter ani. Dr. Bushe removed the tumors, and so successfully, that she shortly married, and had no return of her disease.—*Bushe.*

*Case* 3. Mrs. ——, from childhood had been subject to constipation of the bowels. She menstruated at fourteen, and experienced no derangement of the uterine system until between fifteen and sixteen; then, the discharge ceased for four months, during which time she lost a considerable quantity of blood from hemorrhoidal tumors. With the return of the uterine, the hemorrhoidal flux disappeared in great measure; but the tumors continued and became exquisitely painful, as well as the surrounding parts, attended with frequent painful calls to micturate. At the age of seventeen she married, but so painful was the vulva in May, 1833, four months after her marriage, that her husband had not been able to cohabit with her. Dr. Bushe being consulted, found the lower extremity of the gut beset with hemorrhoidal tumors. The examination gave rise to exquisite pain, extending to the organs of generation, and to exquisitely marked spasm of the sphincter ani. Dr. Bushe removed the tumors, regulated her bowels, ordered a warm hip bath daily, and suppositories of belladonna and opium every twelve or twenty-four hours. In three weeks she had perfectly recovered.

*Case* 4. Mr. —— consulted Dr. Bushe for "neuralgia of the testicles." He had piles, from which the pain extended to the perineum, testicles, bladder and urethra. He was emaciated and worn down by continual suffering and nervous excitement. For fourteen months, he had tried all the most powerful narcotic remedies, as well as iron, bark and arsenic, both warm and cold bathing, exercise, a sea voyage, and so on; but without advantage. Dr. Bushe first regulated his diet and bowels, and then removed the hemorrhoidal tumors, with such advantage, that in a month he had not a remnant of his very painful disease.

e. When we recollect, says Dr. Bushe, the structure and situation of these tumors, we ought not to be surprised, that they very often become inflamed, increase much in size, are attended with great pain, muco-purulent discharge, and disorder of the urinary organs. In this state, provided the tumefaction be great, the patient feels as if there were foreign matter in the rectum, straining ensues and they are prolapsed, now, the sphincter becomes affected spasmodically and presses on their radices, giving rise to great suffering. The inflammation may subside in one, two, or three days, and then these tumors will either recede of themselves, or the patient be able to return them in the usual manner; but it sometimes happens that the sphincter contracts with so much force, as to strangulate them, and cause mortification; an event which generally effects a radical cure, though a few cases are recorded in which the issue was mortal. Of four cases which Dr. Bushe has seen, three terminated favourably, and one fatally.

*f.* It not unfrequently happens, continues Dr. Bushe, that, in consequence of inflammation in these tumors, small abscesses form in them, attended with a discharge of purulent matter from the anus, and more pain and irritation of this part than usual. Such cases are far from being uncommon, and are too often overlooked. To detect these small fistulæ the finger ought to be cautiously introduced, and after a little exploration, a small depression, marking the fistulous orifice, may be discovered on each tumor thus affected. But should this attempt fail, the buttocks ought to be forcibly separated by an assistant, while the patient bears down; then, with a strong light and a probe of a small size, the sinus will be easily found. Dr. Bushe has occasionally seen two or even more of the tumors fistulous.

*g.* Occasionally, these tumours are attacked with ulceration, and in such cases, it generally seizes on many points at the same time, but seldom advances to any great extent. I have, however, seen a case in which three very large hemorrhoidal tumours were one half consumed; and in the twelfth chapter, page 138, I have related another, which is interesting on account of the phagedenic character of the disease. Hemorrhage is sometimes the result of the ulcerative process, as I had an opportunity of observing in the two following cases: Mr. C., a gentleman advancing in life, of full habit, and subject to hemorrhoids for many years, during a salivation, which resulted from the mercurial treatment of a severe fever in the West, was attacked with more than usual uneasiness and purulent discharge from the rectum while at stool. In a few days he began to bleed, and so much did this increase, that he repaired to New-York, and became my patient. He was very low from loss of blood, and distressed in mind. I made a careful examination, and found four hemorrhoidal tumours, one of which was as large as a peach stone, and ulcerated deeply. When he strained, all the tumours were prolapsed, and florid blood issued freely from the ulcerated surface. I removed the tumours, and he soon regained his health. The other case was that of a planter from Louisiana, who arrived here this summer, on his way to Paris, to be operated on; but so low was he when he reached this city, that he felt unable to proceed, and, therefore, sent for me, and had the operation performed. In this case there were several ulcerated points on each tumour, and though they were superficial, the hemorrhage from them was very brisk. Notwithstanding I have seen several cases of ulcerated hemorrhoidal tumours, those are the only ones that were hemorrhagic, and this I am inclined to attribute to the condensation which they generally undergo from repeated attacks of inflammation, previous to the commencement of the ulcerative process." 159.

*h.* Though these tumors maintain their spongy structure for years, yet it occasionally happens that, from constant irritation they become transformed into a semi-cartilaginous mass, being firm, yellow, and nearly bloodless. Dr. Bushe removed some tumors of this kind from a lady.

*i.* Before we quit the consideration of the symptoms and consequences of internal hæmorrhoidal tumors, we must draw attention to some remarks of Mr. Syme's. They are of considerable practical value.

"The existence of bleeding from internal hemorrhoids frequently escapes the observation of the medical attendant, from the patient carelessly overlooking or wilfully concealing it. In females, the delicacy of the sex, which is an additional obstacle to discovering the disease, should excite corresponding vigilance on the part of the surgeon; and whenever there is any ground for suspecting its existence, be the patient male or female, an examination of the bowel in its most protruded state should be insisted upon before giving any opinion of the case. It is also very necessary to beware that the symptoms, especially those connected with the circulation, do not obscure the nature of the disorder, and make it appear to depend on what are really its secondary effects. As an instance of this, I may take the case of a gentleman, about 40, an English commercial traveller, whom I saw last spring with Dr. Alexander. He had laboured long under what was supposed to be disease of the heart, and been treated for this complaint by one of the most eminent provincial physicians in England. His waxy look, bloodless lips, and defective energy, together with irregular action of the heart, certainly afforded considerable ground for this opinion; but Dr. Alexander discovered that there was an internal hemorrhoid, which bled profusely every time the patient went to stool, and I removed it, with the effect of quickly restoring him to health. There is reason to fear that in such cases as this the cause has not only been mistaken for the effect, but may even have been supposed to exert a salutary influence in moderating the violence of its action,—in other words, that the flow of blood from the rectum depended upon disease of the head or heart, and was useful in lessening its force. Such erroneous views may have led to the equally erroneous practice of abstracting blood artificially in these circumstances, the effect of which may be easily imagined." 72.

Perhaps no words of our's could add to the force of these recommendations. Yet we may be permitted to observe that the rectum is probably too little examined, and that descriptions of symptoms relating directly or indirectly to it, are too often taken on trust. The reasons are not hard to guess. An examination is not pleasant, either to medical attendant or patient, and, if the latter is a female, nothing but necessity will justify it, at least in her eyes. Yet if the surgeon consults his reputation and his patient's welfare, he will insist on an examination when positive symptoms are complained of, or when suspicious symptoms not otherwise well accounted for exist. It was but a very few days ago that we saw a patient, who, by the advice of a medical man, had been wearing a truss for prolapsus ani, and whose complaint we ascertained, on examination of the bowel, to be internal piles and ulcer of the rectum. The truss had been ordered without accurate examination.

c. *Tumors at the Verge of the Anus, or "Venous Hæmorrhoids."* Dr. Bushe only takes up the consideration of these tumors at an advanced stage. Their early stage he does not notice, or, if his observations refer to that period, they seem to us to be incorrect. Dr. Bushe considers these tumors as resulting throughout from blood extravasated into the cellular membrane.

Mr. Syme takes a much more correct view of their origin, but, oddly enough, he does not allude to their occasional termination. So, in fact,

Mr. Syme describes one stage, and Dr. Bushe another. Each offers his description as complete, while, in reality, that of neither is so.

Mr. Syme, then, describes this second kind of hæmorrhoidal tumor in its origin. Dr. Bushe exhibits it in its advanced condition.

The lower part of the rectum, says the former gentleman, is supplied with numerous veins lying under the mucous membrane, through which they may be readily distinguished. These vessels in the neighbourhood of the anus are liable to varicose enlargement, and then present the appearance of irregular tumors encroaching on the cavity of the gut. They extend for an inch or two above the anus, but hardly show themselves beyond it, unless the nates are held aside, when they may be seen projecting from the sides of the orifice. They possess a dark colour, smooth surface, circumscribed form, and tense consistence. The veins thus altered are liable to inflammation of the same subacute kind to which the varicose *vena saphena* is subject. In this state they become larger, harder, and excessively painful, especially when in the slightest degree compressed, so that sitting and evacuating the bowels occasion great distress. The blood circulating through them frequently coagulates during such attacks; and if it subsequently undergoes absorption, a spontaneous cure may be accomplished. At other times suppuration ensues in the surrounding cellular substance, and may thus lay the foundation of *fistula in ano*. A discharge of blood also occasionally proceeds from ulceration of the enlarged veins, just as happens in the leg.

This form of the disease has attracted more attention than either of the others, and has even been supposed to be the sole cause of hemorrhoidal swellings. In a slight degree it is certainly very common, and to this extent frequently exists, along with enlargement of the neighbouring textures; but without such combination it rarely attains sufficient size to produce much inconvenience, or attract the patient's notice. The situation of the visible part of the tumors, neither within nor altogether without the sphincter, together with their form consistence, and colour, render their recognition very easy.

Thus Mr. Syme will be observed to place the commencement of these tumors in dilated veins. We have noticed, in the examination of dead bodies, the veins beneath the mucous membrane of the bowel, at the anus, excessively dilated, and forming distinct varices. That it is by enlargement and inflammation of these veins that many hæmorrhoidal tumors begin, we can have no sort of doubt. Yet it is singular that so accurate an observer as Dr. Bushe should overlook, or, rather, deny this circumstance. Take his description.

"The second class of tumours are those situated on the verge of the anus, though I have seen a few cases in which they extended a short way within this orifice, being in part covered with the mucous membrane. They are more or less livid, generally elastic, have an extensive base, and are formed of extravasated blood, which is encysted by condensed cellular tissue, and covered by a few fibres of the sphincter and fine skin of the verge of the anus. I have satisfied myself of these facts, by cutting off the prominent portion of the tumour and then turning out the extravasated blood, in the living body, and by cautious dissection in the dead. Sometimes the blood is absorbed, leaving no trace behind; occasionally, however, in consequence of the first, but more especially of repeated attacks, the superincumbent integuments and surrounding cellular tissue, become hypertrophied, and pendulous flaps or tumours, which in some instances, from the friction they are exposed to, obtain a rough or warty aspect, and become a source of great irritation. It not unfrequently happens, that when there is but one large tumour, it suppurates and then gradually shrinks up.*" 164.

It appears to us that Dr. Bushe has examined these tumors at an advanced stage, and in an aggravated degree, when the vein leading to the coagulum in the original varyx is choked up, and when the varyx looks like an independent extravasation. We are unable otherwise to account for Dr. Bushe's denial of a fact equally obvious in the living and the dead—the not unfrequent occurrence of varices in the hæmorrhoidal veins at the anus.

D. *External Hæmorrhoids.*—Mr. Syme's description of these is much superior to any we find in Dr. Bushe's work. To it, therefore, we devote ourselves. The thin skin, we quote Mr. Syme, which connects the internal mucous and external cutaneous covering at the anus, like the same texture in other situations as the lip and prepuce, is liable to swelling, from distention of the loose cellular substance which lies under it. Any irritation in the vicinity may occasion this; and the derangement once induced contributes to its own increase, by causing protrusion of the affected part beyond the sphincter, when, the circulation being impeded, the tendency to inflammatory engorgement is promoted. A tense red tumour, or series of tumours, may now be seen at the margin of the anus, easily distinguishable from varicose veins in the same situation, by their florid colour, pyriform shape, and more yielding consistence. In other respects the symptoms are nearly the same. The inflammation usually terminates in resolution, but sometimes leads to suppuration, and also, though very rarely, proceeds to mortification. When the engorgement attending the excited action subsides, the distended skin may resume its natural condition completely, but, in general, does so only partially, and remaining relaxed, constitutes a permanent pendulous fold at the orifice of the gut, always ready to resent any irritation, and swell to its former or even a still larger size.

The artificial mode of life which results from the usages of civilized society tends so strongly to the production of hemorrhoidal disease, that few people remain altogether free from it; and this form is the one which it most frequently assumes, often existing independently of any other morbid affection, and very generally accompanying other diseases of the rectum.

E. *Causes of Hæmorrhoidal Tumors.*—Dr. Bushe enumerates them very fully. The catalogue may, at first sight, appear formidable. They are, the structure of the part,—age,—sex,—climate,—period of the year,—hereditary predisposition,—the suppression of other hemorrhages,—habit,—plethora,—other diseases,—passions,—constipation,—pregnancy,—the development of tumors in the pelvis and abdomen,—disease of the liver, pancreas, spleen, lungs, heart or aorta,—obliteration of the inferior mesenteric vein,—tight lacing,—concussion of the abdomen,—the application of bandages to the inferior extremities,—pierced seats,—certain alimentary substances,—stim-

* "I had an opportunity of dissecting a case of this kind, in a soldier, who, died of fever, and I ascertained that the abscess was in the cellular tissue, *not in a vein*."

ulating purgatives,—irritating enemata,—diarrhœa,—dysentery,—prolapsus of the rectum,—ascarides,—external irritation,—stone in the bladder,—stricture of the urethra,—disease of the prostate,—and excessive venery.

Dr. Bushe considers these causes in detail. We need not follow him through each particular, but merely take notice of the leading circumstances.

*a. Age.* "Adults are more liable to hemorrhoidal affections than youths. I have, however, observed a well marked case, attended with hemorrhage, in a boy five years old, who laboured under stone; and another in a girl, between six and seven. Such cases, however, are very rare, first, because in early life, the head and chest are more subject to vascular repletion, than the abdomen; whereas, in mature life, this region is peculiarly susceptible of sanguineous engorgement; secondly, because the venous system is more fully developed in the adult, and the circulation less rapid; and, thirdly, because the bilious temperament and depressing passions pertain, for the most part, to those who have passed the period of puberty. Besides these differences, which are applicable to both sexes, there is another peculiar to females. This is the cessation of the natural menstrual discharge, in consequence of which, especially in plethoric women, the system becomes surcharged with blood. If, under such circumstances, the vessels of the rectum exhale the superfluous blood, we look upon the hemorrhage as a fortunate occurrence, for in this way fatal attacks of apoplexy and other diseases are warded off. 168."

*b. Sex.* In opposition to the prevalent opinion, Dr. Bushe thinks men more prone to hemorrhoids than women.

*c. Hereditary Predisposition.*—Perhaps it is hardly necessary to quote facts in favour of this. Children of hemorrhoidal parents, possess a similar organization, and are, in consequence of it, predisposed to these affections. Many authors have related cases, with a view of illustrating this tendency. M. de Larroque mentions an entire family, amounting to eight or nine in number, who were thus afflicted.

*d. Suppression of other Hæmorrhages.*—Dr. Bushe relates one or two cases in corroboration of this not unimportant fact.

*Case* 1. A gentleman, now upwards of 50, left Ireland several years ago, in consequence of a dangerous spitting of blood, and settled in the South, where he transacted a large commercial business. The expectoration of blood soon subsided; but he was attacked with hemorrhoids, and though the discharge of blood was considerable, he continued to enjoy excellent health. Having made his fortune, and being excessively annoyed with the constant protrusion of the tubercles, when he attempted to walk, he went to Paris, and had them removed by the late M. Dupuytren. He soon recovered from the operation, and returned to the United States, labouring under a determination of blood to the head, for which he consulted Dr. Bushe. He recommended low living, leeching the anus, and pills consisting of the extract of aloes and blue mass. Under this treatment, he experienced great relief; but though a period of three years has since elapsed, he has been compelled repeatedly to have recourse to the same means.

*Case* 2.—"A gentleman, between forty and fifty years of age, from boyhood had been subject to epistaxis, yet enjoyed perfect health. In 1832, he laboured severely some weeks from headache, vertigo and syncope, in consequence of the suppression of the nasal hemorrhage. He consulted me, and I recommended leeches to the schneiderian membrane, and a cathartic, from which he derived no advantage, for the symptoms above mentioned continued, and he became chilly, suffered from abdominal pain, and a sense of weight in the rectum. I now ordered a stimulating pediluvium, and a brisk purgative. He soon felt a desire to defecate, and while on the chair, evacuated quite a pint of blood. Immediate relief followed this discharge, and in the course of the night, he lost about as much more blood, during the operation of his medicine. When I visited him on the following morning, he felt perfectly restored, and wished to go to his counting-house, a request which I, of course, did not comply with. He was rapidly restored to his wonted condition, in which he still continues, through the medium of a regular hemorrhoidal flux, which seems to have supplied the place of the epistaxis." 172.

e. We perceive no necessity for pursuing these details farther. Our readers need not be told, for example, how constipation or pregnancy may lead to hæmorrhoids. But, on a review of their causes, Dr. Bushe divides these affections into constitutional and accidental, and he is inclined to lay some stress on the distinction.

"When they are constitutional, there is an hereditary predisposition, they are generally of long standing, and the attacks, which are frequently periodical, occur independently of local and accidental causes. The relief resulting from the attack, the quantity of blood effused, the existence of tumours, the intensity and duration of the pain, the age, season, climate and habits, should be taken into account, when determining the nature of the affection.

In the accidental affections, the hereditary predisposition does not exist, and neither age, season, climate, nor habit are concerned in their production. The paroxysms are not periodical, and they afford but little relief, unless the evacuation has been preceded by violent congestion of the vessels of the rectum. A local cause easily determined, produces and prolongs the attack; but the best proof of the accidental nature of the disease, is the absence of the symptoms denoting a fluxionary movement, and finally, the presence of but a few local symptoms." 176.

*Enlargement of the Hæmorrhoidal Veins.*—Before we proceed to the treatment of hæmorrhoidal tumors, we ought to mention that Dr. Bushe devotes a separate chapter to "Enlargement of the Hæmorrhoidal Veins." As this bears on the points to which we have referred a few pages back,* we think it right to put our readers in possession of Dr. Bushe's sentiments, on a matter of some pathological interest, if not importance. We must quote his words:—

"In the chapter on hemorrhoidal affections, I gave such a description of the tumours which result from a determination of blood to the rectum, as I was warranted, from the examinations I had made of them. Whether they had their origin in diseased veins, I did not, nor do I now, pretend to determine. Some one possessing more leisure than falls to my lot, would do well to renew the investigation, freeing his mind, in the first instance, from the plausible theory of their venous origin, and recollecting also, that a morbid structure may not have the same identical arrangement, from the commencement.

Slight dilatation of the hemorrhoidal veins is very common, especially in persons subject to enlargement of the veins of the inferior extremities, and in such persons, the portal veins are generally more ample, and have thinner tunics, than

* Vide pp. 307—8.

in those who are free of this infirmity. These facts I long ago satisfied myself of.* I must say, however, that the dilatation of the hemorrhoidal veins, which I have seen, bore no resemblance, whatever, to the hemorrhoidal tumors; nor did it appear to me, that the dilated veins were undergoing any structural alteration, which would lead to the supposition that they were about to be converted into hæmorrhoidal tumours. In plate iii. fig. ii, is represented the anus of a gentleman, who laboured under excessive dilatation of those veins. The appearance of the disease is certainly very different from that delineated in plate iii. fig. i. and in plate ii. fig. i; the first representing a case of internal, and the second, a case of external hemorrhoidal tumours. The gentleman from whom the drawing alluded to was taken, never lost any blood from the anus, and only experienced inconvenience from the impediment created by the venous mass, to the evacuation of the feces. The dilated veins could be easily felt, not only through the skin, but also through the mucous membrane, even above the edge of the internal sphincter.†" 198.

We would observe, in reference to these remarks of Dr. Bushe, that we have seen decided varices of the hæmorrhoidal veins *at* the anus, in dead bodies—that we have seen a dilated hæmorrhoidal vein lead to a small tumor, apparently, a varyx, containing solid coagulum *at* the anus—and, finally, that the analogy of the veins of the extremities would render it at least probable that hæmorrhoidal tumors may frequently be dilated veins, filled with coagulum, and more or less inflamed. The question is, however, a more open one, than many surgeons may, perhaps, have thought it.

*Treatment of Hæmorrhoidal Affections*.—We shall describe the treatment of hæmorrhoidal congestion, and of its consequences, very nearly in the order and in the circumstantial manner, in which we described those conditions themselves.

A. *Congestion*. When symptoms denoting repletion of the vessels of the rectum exist, we ought to direct the bowels to be evacuated with castor oil, or some other mild cathartic, then, a dozen of leeches to be applied to the anus, and after they have been removed, a warm hip bath. This may or may not produce the hæmorrhoidal discharge. In either case, it usually relieves the congestion.

B. *Hæmorrhage*.—When moderate it requires no special measures. We

---

* "See an Essay on Phlebectosis, by George Bushe, M. D., Medico Chirurgical Bulletin, vol. i. p. 230. New York, 1831.

† "M. Petit relates a case, in which the patient sunk under hæmorrhage from the rectum. On dissection, he says, "je trouvai le foie peu gouflé, mais durs les veines mésentérique, spléniques et autres, qui forment la veine—porte, étoient considérablement dilatées, parce que le troue étoit comprimé, non par le volume, mais par la dureté du foie; les veines hémorrhoïdales, depuis l'S du colon jusqu' au sphincter de l'anus, étoient variqueuses, crevées et ulcérées dans l'interieur du boyau; les bords de plus d'une trentaine de ces ulceres, le boyau même, dans presque toute son étendue, etoiént durs et calleux." (Traité des Maladies Chirurgicales, tome ii. p. 74—5—6. Paris, M.DCC.XC.) Petit, who considered hemorrhoidal tumours as varices, employed this remarkable case in illustration of his views, and none of his followers have failed to bring it into their service; but with what justice, I leave the reader to decide."

have found small quantities of cold water thrown up as an injection *after* the morning stool, of service. When the hæmorrhage passes a certain point, it becomes a substantive evil of consequence. The bowels should be regulated by the lenitive electuary. The Ward's Paste may be given three times daily, in doses of a drachm. Some persons become feverish and uncomfortable under the use of this medicine. Dr. Bushe says that, in such cases, four ounces of lime-water ought to be injected into the rectum every morning and evening, and retained as long as possible. When the patient leads a sedentary life, he should take exercise, by which the secretions will be increased, and the circulation equalized. Dr. Bushe knows a studious gentleman, who suffers much from the hæmorrhoidal flux in the Winter, but in the Summer, when he travels, the bleeding ceases. The diet ought to be plain and moderate.

If the hæmorrhage grows alarming, Dr. Bushe advises the following means:—ice to the perineum and sacral region; sinapisms and ligatures to the upper extremities; cupping-glasses, with or without scarifications, over the scapulæ; diluted sulphuric acid, or acetate of lead with opium internally; and, injections of ice water,—spirits of wine,—port wine,—a solution of alum, of sulphate of iron or copper in a decoction of oak bark,—tincture of the muriate of iron and water,—or, the decoction of bistort, tormentil, pomegranate, nut galls, &c. Dr. Bushe makes no mention of a quack remedy—Ruspini's styptic. Yet it has occasionally *seemed* to be of much service in the hæmorrhages. There are few cases in which we are more likely to over-rate the value of medicines, than we are in cases of hæmorrhage. They so frequently stop from the action and influence of the natural processes, that medicines often get unmerited credit. If Ruspini's styptic sometimes answers, we have seen it much more often fail.

"Some authors," continues Dr. Bushe, "have recommended bleeding in the arm; but I cannot add my assent to this practice, for though I am ready to admit that it diminishes the nervous agitations, renders the disposition to metastasis less easy by emptying the vessels, and tends to draw the blood to the superior part of the body, I am disposed to think that a patient reduced by the hemorrhoidal flux, has got no blood to spare. In the early stages of the flux, when the vital forces are exalted, this objection will not hold good, but at such time phlebotomy would be a highly improper remedy, for, as I have said before, it is only when the hemorrhage is profuse that we are justified in meddling with it.

When the hemorrhage has continued so as to exhaust the patient by slow degrees, and has assumed more or less of a passive character, we ought to administer the sulphate of quinine and sulphuric acid, or some chalybeate preparation, with great caution; but most advantage will be derived from sea bathing. I have seen a few cases, in which much improvement took place during a course of ferruginous waters.

If the bleeding proceeds from tumours, they ought to be removed." 178.

c. *Internal Tumors.*—When the tumors are extremely painful, Dr. Bushe recommends their being anointed three or four times daily with an ointment of opium. The application of a stream of cold water has been found useful. When the sphincter is spasmodically contracted, the ointment of belladonna is of service.

"Should the tumours be inflamed, leeches ought to be applied to the surrounding parts, and followed by tepid cataplasms. Some authors have recommended scarifications, but, I cannot approve of this practice; firstly, because I have seen much annoyance, and never any good, arise from them; and secondly, because the principle upon which they have been recommended is erroneous, viz.: that as piles are dilated veins, their puncture ought to afford much blood, and thus disgorge the vessels of the rectum. When they descend, and the surrounding parts are relaxed, we may advantageously use the ointment of galls. In consequence of pain, it may be advisable to add opium, or of spasm of the sphincter, belladonna to this ointment; and should there be ulceration and fungous asperities on their surface, the super-acetate of lead will prove a useful addition, in the proportion of half a drachm, or even a drachm, to an ounce.

Where the spasm of the sphincter and pain did not forbid it, I have ordered half a drachm of the sulphate of zinc, in half a pint of water, to be injected every morning after defecation, and in the evening a steel bougie to be passed a few inches into the bowel, and kept so for half an hour. This plan has in some instances answered very well, and on the whole, appears to me much more useful than it was esteemed by those who first tried it." 180.

If, in spite of all these means, the tumors continue to give rise to severe symptoms, or to hæmorrhage, and if, on mature consideration, there is nothing in the local or the general circumstances, deemed calculated to render an operation unadvisable, this step should be resorted to. We say, on mature consideration, for this operation is, after all, not free from immediate or remote hazard, and should not lightly be undertaken.

There are two leading modes of performing it;—by excision—or by the ligature. The dispute on the comparative advantages of the two methods has run high. The opinion of the profession in general may be safely said to be now settling down to the superiority of the latter. The experience of both the authors before us leans to the same side, and to give greater weight to a probable and general conclusion, we shall quote individually their sentiments and practice.

Some surgeons, says Dr. Bushe, prefer excision, and others the ligature. Those who advocate the former, say that there is no danger of hæmorrhage, that it is more readily executed, attended with less pain, and followed by a more rapid recovery, than when ligatures are applied; but above all, that it is entirely free from phlebitis, tetanus, and peritonitis.

Dr. Bushe then takes up the argument, and thus weighs the reasoning on the one side and the other.

"That it," (excision) "is more easily and quickly executed, I am ready to admit, though it cannot be denied that the operation by ligature is simple enough, and far from being tedious. Perhaps, also, it may be somewhat less painful, but in this respect there cannot be much difference, when the ligatures are properly applied. That it is followed by a more rapid recovery, I deny. As to the occurrence of phlebitis, I have never seen a case, nor am I acquainted with one on record. The dread of this consequence has arisen in the minds of some French authors, because they set out with the preconceived idea that these tumours were varices, and then, reasoning from analogy, they arrived at a conclusion which is not tenable. An author, therefore, instead of quoting cases of inflammation of the veins of the leg, from ligature of the saphena, to illustrate the consequences arising from tying hemorrhoidal tumours, ought to have read some of our English authors, who, though they agree with Briquet, Danse, and others, as to the occurrence of inflammation in some cases of ligature of the saphena, have, at the same time, demonstrated that the extremities of this vein are much less liable to become inflamed than its trunk, in consequence of external violence. Mr. Kirby has given a case in which tetanus followed the operation, and in the same manner, I could cite two fatal cases of this dreadful disease, one of which succeeded to a thorn in the heel, and the other followed a very slight abrasion of the skin. It is very much to be regretted that the opponents of the method by ligature have not, like Mr. Kirby, quoted cases, instead of making assertions: such a paucity of facts, therefore, is not likely to have much weight with an impartial surgeon. As to peritonitis, I know of no case on record *in which it was proved by dissection*. Indeed, the occurrence of this disease, as a consequence of the tying piles, was stated by Petit, who related two cases, in which, after the operation, the patients were seized with symptoms resembling those of strangulated intestine, to wit, nausea, vomiting, hiccough and abdominal pains. One of these patients recovered, and the other died on the second day, but as no examination of the body was made after death, we are not justified in drawing any conclusion from it. That it was a case at all fitted for operation, we do not know, and, however probable, it may be questioned, whether the fatal attack, whatever it may have been, was the result of the operation.

That excision is not likely to be attended with hemorrhage I deny, for I have performed the operation several times, and after it, have had to tie up arteries, plug the rectum, and in one instance to apply the actual cautery. Indeed, I so nearly lost two patients, that when left to my own choice, I no longer have recourse to this operation. In the cases I have operated on, the hemorrhage has never been alarming during the operation, but in one instance, and in it, I was compelled to make firm pressure with the two first fingers of my left hand, for a considerable length of time, a procedure which appeared necessary to prevent a most frightful hemorrhage. Generally, however, after these operations, the hemorrhage does not occur for a few hours, then, the patient who may have been perfectly comfortable, becomes anxious, restless, and is seized with rigors, spasms of the extremities, cold perspiration, sickness of the stomach, swelling and tension of the abdomen, particularly in the left iliac fossa, and colic pains. His pulse becomes small, frequent, and irregular; his respiration anxious; his countenance pale; he is vertiginous, and faints. All this time, the blood is accumulating in the colon, and he may die without discharging it; but frequently the tenesmus is so great, that he goes to stool, evacuates large clots of blood, faints, and sometimes dies. More commonly, however, the discharge, if it takes place in the recumbent position, brings relief; but, after some time, the hemorrhage returns, and in this way some patients have died.

From what I have now said, it will appear that excision of hemorrhoidal tumours, is far from being a safe operation. Indeed, we cannot free it from the danger of hemorrhage, unless we touch the cut surfaces with the actual cautery, as recommended by Dupuytren, and I have no hesitation in saying that this is a barbarous proceeding, and one that ought not to be adopted, since we possess a certain and comparatively safe remedy in the ligature." 184.

Mr. Syme's sentiments are substantially the same as those of Dr. Bushe. He admits that excision is the quickest and the easiest mode of removing the tumors, but it is exposed to the fatal disadvantage of being liable to give rise to serious hæmorrhage. Sir A. Cooper, it is well known, has related several disastrous cases. Other surgeons have not been so candid, but it does not follow that they have been more successful.

"Before," says Mr. Syme, "my own views were settled as to the best means of treating the disease, I on one occasion cut away an internal hemorrhoid, which was partially protruded, and found it necessary to employ manual pressure for several hours to restrain the bleeding that followed. In another case of the same kind, I succeeded in securing the vessels by ligature." 73.

Mr. Syme, then, is opposed, as the majority of surgeons are, to the operation of excision. It is not likely, that, when patients are properly acquainted with its dangers, they will strongly insist on its performance. Yet it is possible that, under some combinations of circumstances, excision may be not only proper but necessary. The surgeon should be aware of the manner of performing it. Dr. Bushe details it.

The bowels having been gently moved with oil or an enema, the patient should sit over warm water, and strain until the tumors are prolapsed; then, placing himself sidewise on a couch opposite the window, with his knees drawn towards the chin, an assistant separates the buttocks, while the surgeon with a polypus forceps in the left hand, and a long curved scissors in the right, seizes and excises the tumors one by one, taking care not to include any of the surrounding mucous membrane.

Bleeding is the thing to be apprehended. The surgeon should, therefore, be prepared for it. The directions for its arrest supplied by Dr. Bushe are the following.

If the bleeding be profuse, the operator should introduce his finger, and desire the patient to contract the sphincters as closely as possible, so as to compress the bleeding vessels. In a short time, the finger may be withdrawn; but, it will be prudent to elevate the hips, and apply small bags of ice to the anus. If the hæmorrhage recurs after a few hours, the anus ought to be dilated with a speculum, and if possible, the bleeding vessel or vessels secured with ligature; but, if we cannot accomplish this, the cut surfaces should be touched with the actual cautery. Some patients, however, will not hear of this means of security, and under such circumstances we are compelled to resort to compression.

"With a view to accomplish this in the most unexceptionable manner, I would recommend the use of the following instrument, which I had constructed for suppressing hemorrhage after lithotomy. This instrument is seven inches long, tubular, about as thick as a swan's quill, terminated with a button at one end, to facilitate its introduction, and with a stop cock at the other. One inch from the stop cock, and half an inch from the button, there are two projecting rings, and on the proximal side of the distal ring, the tube is perforated by a number of holes. Finally, a portion of intestine is bound by means of waxed silk, on the tube, behind the rings. This instrument should be introduced and then inflated. In some little time we can let off the air, and withdraw the instrument, provided the hemorrhage has ceased; but, if we find that it returns on the removal of the pressure, we must again inflate the intestine." 186.

Petit introduced a sort of bag into the rectum, and stuffed it, when in the bowel, with lint. By this means, he could compress the parietes of the gut, and, by unstuffing the bag, withdraw the plug when he pleased. Dessault made use of a nearly similar contrivance. Dupuytren makes mention of another, similar in principle, and occasionally put in practice by other surgeons;—it is the introduction into the rectum of a pig's bladder which is subsequently stuffed with lint. We are inclined to think that the stuffing of some bag, be it bladder or what not, with lint or tow, is more likely to answer than its inflation with air, in the mode advised by Dr. Bushe.

Mr. Syme alludes to another plan which has been proposed, but is open to the objections that he urges. The proposal, in question, has been to transfix the base of the protruded part with pins, to prevent the raw surface from being drawn within the sphincter until the bleeding ceases, or is arrested by ligature. But it is to be feared that the hæmorrhage, though prevented so long as the part was kept tense by the pins, might occur after their removal, unless they were allowed to remain until the orifices were sealed up with lymph, which could not be done without the risk of exciting inflammation and constitutional disturbance.

After the operation, the patient ought to be confined to arrow root, barley water, and such nourishment. If he suffers pain, or is restless, it will be necessary to administer morphine, and mental excitement should, if possible, be prevented. On the third day an enema, consisting of gruel and oil, should be given, and repeated every second day until the parts heal.

Dr. Bushe does not seem to us to dwell sufficiently on the necessity for clearing out the bowels well before the operation, in order that they may not be disturbed after it. If they are properly evacuated beforehand, a dose of opium or of morphine may be given immediately afterwards, with the effect of soothing pain, relieving spasm, and diminishing the chance of mischief.

We turn to the operation by the ligature, admitted by most surgeons to be much the best.

If, however, the operation by the ligature does not carry with it the dangers of hæmorrhage, may it not have some of its own? It has been alleged to entail such danger in inflammation spreading from the strangled parts, and either terminating fatally, or causing extensive suppuration and sloughing in the neighbourhood of the anus. Mr. Syme thus meets and disposes of this objection.

"The seeming resemblance," he says, "between the condition of an internal hemorrhoid, to which a ligature has been applied, and a strangulated hernia, makes it appear likely that this effect would follow the operation; but experience teaches, what a more careful analysis of the cases would lead us to expect, that the bad consequences thus anticipated do not really present themselves. In a strangulated hernia, the circulation of the protruded parts is not entirely obstructed, but merely impeded, so as to cause inflammation, with its usual local and constitutional symptoms, aggravated by the importance of the affected part; while a hemorrhoid subjected to the ligature is completely detached from any share in the vital action of the system, which, consequently, cannot be influenced by its condition. Accordingly, however similar the two cases may appear at first view, their results prove very different; and I feel warranted, after very extensive employment of the ligature, to state, that it may be used without the slightest risk of serious or alarming inconvenience.

In order to account for the bad consequences which Mr. Copeland and others have related as occasionally attending the use of the ligature, it will be sufficient to remark, that if the threads are not drawn tight,—if such large portions of the morbid texture are embraced by them as to prevent the degree of compression requisite for preventing altogether the circulation through the tumours,—or if the whole of the disease is not included, disagreeable effects may not improbably ensue. Sir A. Cooper has advised that the ligatures should not be drawn tight, with the view of lessening the pain caused by them. But, with all deference to his high and justly esteemed authority, I feel no hesitation in stating, that though the suffering of the patient may in this way be rendered less severe in the first instance, it will ultimately be much greater, as well as more prolonged, and attended with more danger of spreading inflammation, than if the strangulation had been completed at once. To obviate this objection, it has been proposed to cut away the tumours, immediately after they are tied, close or near to the knot, which method, it is obvious, must be attended with another danger, since the ligature, when thus left unsupported, will be apt to slip off, and permit the vessels to bleed. If the threads are drawn tight they will not so readily quit their hold; but in this case no advantage can be derived from removing the strangulated parts, which then cease to maintain any living action, and very soon collapse into the form of flaccid bags." 78.

There can be no question that the operation by the ligature is, on the whole, a very safe one. Yet no operation is absolutely safe. We saw a patient die from abscess in the pelvis after the tying of internal hæmorrhoids. The ligatures were drawn very tight. Dr. Bushe adds his testimony to the safety of the operation in general. He says he has performed it upwards of a hundred times and never seen a bad symptom follow it.

Mr. Syme continues:

"I thought at one time that the best plan of proceeding with the ligature was to include at first only a part of the disease, with the view of avoiding any risk of exciting more irritation than the part or patient could safely bear; but I am now persuaded that by doing so much more pain and danger of undue excitement are occasioned than by the summary process of tying all the tumors at once. In illustration of this I may mention the case of an eminent provincial practitioner whom I attended with Dr. Abercrombie. He had long suffered from the bleeding of internal hemorrhoids, and was at length reduced to a state of extreme exhaustion. From being a strong muscular man, he had become a feeble emaciated invalid, unable for any exertion of body or mind, with the waxy look, frequent small pulse, and headache in assuming the erect posture, which characterize the state arising from continued depletion. As the tumours were large and numerous, I commenced the treatment by tying one of the smallest, with the view of ascertaining what degree of freedom might be used with the remainder. The ligature separated at the end of two days, but the other excrescences swelled and protruded from the anus to the excessive distress of the patient, who described his suffering as intolerable, and alarmed the neighbours by his cries. As his pulse suffered little alteration in frequency or hardness, and his belly continued free from pain, no great apprehensions were entertained as to the result. The inflammation accordingly did not extend beyond the limits of the diseased growth, the whole of which mortified and sloughed off, leaving the patient completely freed from his complaint, though at the expense of much more suffering than had been anticipated.

It is not difficult to explain why a partial operation should produce such effects. The morbid texture of the hemorrhoidal tumours, like all other formations not entering into the original constitution of the body, being hasty and violent in its disposition to excited action, readily inflames when injured, and suffers more acutely than the natural textures. The slightest excitement is apt to make it swelled and painful, and when it is partially subjected to a tight ligature, inflammation so intense as to destroy its vitality may be occasioned, while, if the whole be included the separation takes place, not indeed without some uneasiness, but certainly without any of a serious or alarming character. On the same principle any operation attended with local irritation in the neighbourhood of internal hemorrhoids, is apt to be followed by troublesome consequences from their excitement. A gentleman came under my care for *fistula in ano* with this complication. I advised that both complaints should be remedied at the same time, to prevent the irritation caused by an operation for one of them, from injuriously affecting the other. The patient, however, persisted in requiring the fistula to be cut by itself in the first place, which was done, and followed by a very distressing paroxysm of the hemorrhoidal disease. He returned to the country to recruit his health, and came back some weeks afterwards to have the excrescence removed. Another patient came lately above a hundred miles' distance to be operated upon for fistula, and made no mention of any other ailment. I performed the necessary incision, and a day or two afterwards was surprised to see a large internal hemorrhoid protruding from the wound. He then told me that he had long suffered from bleeding piles; and I expressed my regret that this communication had not been made sooner, as both diseases might have been remedied together, with less inconvenience than he was then subjected to. It happened fortunately that the inflammation proved so intense as to destroy the tumour, which sloughed off, so that the recovery was completed without any farther operation, but certainly, as in the last case, with much more pain and confinement than if the hemorrhoid had been tied when the fistula was cut. Still pursuing the same principle, when any pendulous folds of skin are observed to surround the anus in a case of internal hemorrhoids, I should advise them to be removed with the scissors at the same time the ligatures are applied, lest they inflame and prove troublesome in consequence of the neighbouring irritation." 82.

Dr. Bushe coincides with with Mr. Syme in advising the removal of all the tumors, and not in their extirpation by instalment. He has not seen any bad consequences from the wholesale operation. But when he wishes to *moderate, not to check* the hæmorrhage, he only removes one or two tumors.

Dr. Bushe, too, agrees with the majority of surgeons, in advising that the ligatures should be tied tight. He considers the dread of pain as fanciful.

Dr. Bushe has invented some instruments for the purpose of facilitating the performance of the operation. He declares that he has used them with much satisfaction. Those instruments are—a forceps for seizing and bringing forward the tumors, needles of different sizes, needle carrier, and forceps for removing the needles.

The forceps is six inches in length, shaped like that used for dissection, except that its blades, which are gently curved, terminate in two prongs, one sixteenth of an inch apart, and bent inward for one quarter of an inch, so as to overlap each other. The blades are furnished with a graduated clasp, about two inches from their extremities, so that when pressed together, they remain shut, and consequently keep their hold.

The needles vary in length, from half an inch, to an inch, but are of the same breadth and curve. In each the hole for receiving the ligature is about a quarter of an inch from the point, and the other extremity, for nearly the same extent, is reduced one half; so that it may fit into a socket intended for it, in the needle carrier.

The needle carrier is eight inches in length, and formed like a dissecting hook, save at its distal extremity, which is considerably thicker, less curved laterally, with a socket in its extremity for receiving the needle, and a bracket on its most convex part for supporting the ligature. Besides these differences, it is also gently bent forwards.

The forceps for withdrawing the needle is like the common dressing forceps, except that the blades are curved, so as only to touch at their extremities, which are so scooped out as to accommodate the needle.

Such are the instruments used by Dr. Bushe. The following is his mode of using them:—

The patient being placed in the same position, with the tumors prolapsed, and the buttock elevated by an assistant, as in the operation for excision, the operator seizes the largest tumor with the forceps, and bears it downwards; then, with a needle, long or short, according to the size of the tumor, armed with a strong double ligature of three twist silk, and secured in the needle carrier, he transfixes the centre of the tumor. All this can be expeditiously accomplished, without entangling the needle in the surrounding parts; because, the convex portion of the needle carrier, being alone opposed to the prolapsed parts, it pushes them out of the way without injury, and thus makes room for the ascent of the needle, so that we can see precisely where to enter this point. The needle should now be seized with the second forceps, withdrawn, and cut off. Each half of the tumor being tied as firmly as possible, all of it, save a small portion in front of the ligatures, ought to be cut off with a curved scissors. The other tumors should be treated the same way in succession, and finally, the ligatures being cut off, half an inch from the knots, they ought to be returned, together with the protruded membrane, and remnants of the tumors, within the sphincter.

It will be observed that Dr. Bushe is in favour of the removal by excision, of a large portion of the pile, after it has been tied. Mr. Syme imagines that the ligature will then be apt to slip. But it is clear that if the base of the pile has been transfixed, and either half tied separately, the ligatures cannot slip unless the whole pile is removed, which it should not be. We have tied and we have very often seen piles tied in this way, and we must confess that we have never seen the ligatures slip off. Nor, we repeat, is it possible that they *can* do so, unless the surgeon actually cuts down to them.

Mr. Syme's method of operating differs from that of Dr. Bushe, on account of his not employing any peculiar instruments. His mode is this:—

When the operation is to be performed the patient should take a dose of castor oil, so as to evacuate his bowels previously to it, as they had better not be moved for forty-eight hours afterwards. The hæmorrhoids having been fully protruded by a sufficient degree of straining, the patient either stoops forward, resting with his arms on a chair or table; or if a female, lies on one side with the limbs drawn up, so as to expose the parts concerned. The surgeon then introduces the fore-finger of his left hand within the sort of ring which is formed by the morbid growths, and, keeping it there as a guide, transfixes their roots in succession with a needle and double thread, directed from without inwards through the centre of each close to the base. The ligatures, which should be waxed silk of proved strength, are next to be tied as tightly as possible, each of course including the half of a tumor. Their ends are then cut away as near to the knots as may be, without endangering their security; and the protruded parts are lastly pressed gently back within the sphincter.

Neither Dr. Bushe nor Mr. Syme takes notice of a mode of rendering the application of the ligature more effectual, which, trivial as it may seem, is not useless. The ligatures having been tied in a single knot as tight as possible, the pile should be punctured. Its fluids escape, it shrivels, and the ligatures then admit of being drawn tighter, and the knot doubled as usual. When this has been done a portion of each pile may be snipped off, the ends of the ligature removed, and the protruded parts returned within the sphincter.

The treatment of the patient subsequently to the operation is, of course, not to be neglected.

Mr. Syme remarks, that the pain resulting from the application of the ligature is generally considerable, especially at first, but commonly subsides gradually in the course of a few hours.

"Want of sleep," he continues, "is frequently one of the effects produced, and is sometimes so distressing and prolonged as to excite serious alarm. It is accompanied with nervous excitement, rendering the patient restless, more or less incoherent in his ideas, and wild in appearance. The pulse is seldom much affected, and when it does suffer disturbance, merely becomes quicker without any of the hardness which denotes an inflammatory state of the system. The bowels are constipated, so as not only to cease evacuating their contents spontaneously, but to require laxatives of greater power than is sufficient in ordinary circumstances. Difficulty of making water, sometimes amounting to complete retention, and requiring the catheter to be introduced, very frequently occurs, but seldom continues beyond the first twenty-four hours. In two cases I have found it last for nearly a fortnight. When the patient goes to stool a day or two after the operation, there is either no protrusion at all, or a much smaller one than formerly, and in general no bleeding. Little inconvenience is experienced after the unpleasant effects immediately consequent upon the operation have subsided, until the ligatures separate, which is usually about the end of a week; when a painful feeling is often complained of in the raw surface left by the sloughs, and a little blood is occasionally discharged along with the evacuations. Soon after this the irritated parts regain their natural condition, and all the disagreeable symptoms which proceeded from the disease, as well as those caused by the operation, completely disappear." 86.

He recommends that, an opiate, containing thirty or forty drops of the solution of muriate of morphia, should be administered to the patient if he complains of pain, and be repeated from time to time if it continues severe, or a somewhat larger dose may be injected into the rectum with a teaspoonful or two of warm water. Fomentations may at the same time be applied to the anus. And if, notwithstanding the use of these means, much suffering is still experienced, the hip-bath of poppy-head decoction should be employed. The retention of urine if slight may be relieved by giving the *Spiritus Ætheris Nitrici*, or the camphor mixture; and if more obstinate, will require the catheter to be introduced occasionally as long as it lasts. The patient should restrict himself to the antiphlogistic regimen, and drink freely of simple diluents, such as barley-water or linseed tea, to lessen the acrimony of the urine. He should also confine himself to the horizontal posture until the ligatures separate.

Dr. Bushe prescribes a dose of morphine after the operation—a warm bath, if requisite—on the third day, an emollient lavement, and, after its operation, a small opiate enema—the patient now to lie on a couch, to take more substantial nourishment, and to walk about his chamber—on the fifth day, the enemata to be repeated, and the patient to walk out, if the weather is fine. If the ligatures do not come away in a week, they should be pulled gently, daily, till they separate. After their removal, the anus may be besmeared with diluted acetate of lead ointment, or four ounces of a weak solution of the sulphate of zinc may be injected three times a day, and retained as long as possible.

We must say that we prefer Mr. Syme's after-treatment to that of Dr. Bushe. We cannot deem it prudent to direct the patient to walk out, before the ligatures have separated. The horizontal posture is the best until their separation has occurred.

D. *Venous Hæmorrhoids.*—Our readers will remember Dr. Bushe's opinions on the nature of this form of hæmorrhoidal tumor. He believes it to consist of blood extravasated into the cellular tissue, and enclosed in a cyst, formed by that membrane condensed. His treatment is essentially founded on this view, is simple, and, he says, successful.

"When they are not attended with much pain, the horizontal position, low living, gentle cathartics, emollient fomentations and poultices, will be sufficient for their removal. If, however, the pain be considerable, in addition to the means now recommended, we ought to apply a few leeches; but, should there be but one large and elastic tumour, the better practice will be to lay it freely open, and then, with the scooped extremity of a director, turn out the clotted blood. This operation never fails to give relief, and prevents the formation of matter. Some years ago, I attended a Scotch gentleman, who had just arrived from the West Indies, and had one of these tumours as large as a hickory nut, on the verge of the anus, extending for half an inch within the sphincter. I ordered him castor oil, forbid all nourishment except gruel, and enjoined the horizontal position. In the evening, he had not improved, and therefore, I recommended leeches, fomentations and poultices. On the next day the leeches were repeated, but notwithstanding, the tumour suppurated. About three months since, a full habited merchant, just arrived from Rio, went to shoot on Long Island; but was compelled to return to town, in consequence of considerable swelling of the side of the anus. He sent for me, and on examination, I discovered just such a tumour, as that under which the Glasgow gentleman laboured, save that it was rather larger. I immediately laid it open, and turned out the coagulated blood. So sudden and perfect was his relief, that on the same day, without my knowledge, he dined out, and took his wine as usual. The relation of these two cases, will show, I hope in a pretty clear light, the advantage of incision, when these tumours are large, tense, and painful.

When pendulous flaps of integument remain after the absorption of the blood, they ought to be seized with a polypus forceps, and removed with a curved scissors; otherwise, they will entangle the secretions, and in this way give rise to irritation, and finally degenerate." 195.

Mr. Syme is opposed to any operation for these tumors. Believing them to be venous he has before his eyes the fear of venous inflammation. The fears of Mr. Syme contrast with the operations of Dr. Bushe, and the good effects of the boldness of the latter would appear to prove an overcharged apprehension in the former. Yet it is only fair to him to quote his sentiments.

In regard, says he, to the treatment, the tendency of the venous tissue to resent irritation forbids any operation; and excision as well as puncture, which have been recommended, should both be carefully avoided, lest they excite inflammation of the enlarged vessels, and give it the unmanageable character which distinguishes it when of traumatic origin. Soothing measures are the most useful, such as rest in the horizontal posture, gentle laxatives, as castor oil, injections of tepid water into the rectum, and the hip-bath. When the symptoms are severe, leeches may be placed round the anus, opiate injections should be employed, and lotions, containing acetate of lead with opium, applied to the inflamed parts. By these means the paroxysm is subdued in the course of a few hours, or days at the farthest; and by care afterwards in guarding against the causes of excitement, future attacks may either be prevented or rendered less distressing.

E. *External Hæmorrhoids.*—Every one admits that when these require an operation, excision is the proper one. They may easily be snipped off with curved scissors. The blades of the scissors should be directed from the circumference towards the centre of the anus, in order to get at the root of the tumors without taking away any sound skin. The best time for the performance of the operation is when the hæmorrhoids are in a quiescent state; and it should always be insisted upon when they are present in a case requiring any other operation, since unless removed previously, or at the same time, they would be apt to suffer from the irritation, and, by adding the complication of inflamed piles, greatly increase or prolong the patient's sufferings.

Mr. Syme remarks, that while the hæmorrhoids are suffering from inflammation, excision may still be practised, and it should be resorted to if the patient is willing to endure the pain that attends cutting in this state, in order to get speedily relieved from the complaint. If it be thought better to delay the radical cure until the parts get into a condition more favourable for its easy performance, the same soothing means that have been already mentioned as proper in the cure of inflamed venous hæmorrhoids should be employed.

Mr. Syme alludes with becoming reprobation to the proposal of tying external hæmorrhoids—and with contempt to such applications as the unguentum gallorum, &c. The best palliatives, he says, are attention to regimen, the use of gentle laxatives, such as sulphur with cream of tartar, and Ward's paste, which in all diseases of the rectum attended with relaxation has often a remarkably good effect.

The use of the cold water lavement has always seemed to us more effectual than any other single, or even than many other remedies. The mode of employing it which we have found best has been, to direct the patient to throw up a pint to a pint and a half of cold water after breakfast, about five minutes before a stool is sought for. When this has been voided the patient should throw up from two to four ounces of water and retain it. This and the regular use of the cold water bidet, have, in our experience, been highly serviceable in most cases of hæmorrhoids, external or internal.

We would say a word on the Ward's paste. It has always appeared to us that this does not suit persons whose bowels are disposed to be confined, and who are apt to be feverish. We have found the paste disagree with such persons greatly, however combined with laxatives, or other means calculated to ward off pyrexia.

F. *Mucous Discharge.*—If this is the direct result of inflammation, it ought to be treated on general principles. When chronic, and conjoined with tumors, *they* should be removed. The bowels should be regulated by means of gentle aperients, and appropriate tonics, with exercise, pure air, &c., should be prescribed. The local application of astringents may be useful, and the nitrate of silver is of service.

This completes our account of hæmorrhoidal affections, an account which, however long, is not longer than is commensurate with the practical importance of the subject. We proceed to—

### II. Prolapsus of the Rectum.

A. *Forms of the Disease.*—Dr. Bushe observes that there are two. In one the mucous membrane is alone prolapsed; whereas, in the other, all the coats of the rectum come down. The first is by far the most common, in consequence of the great extent and loose connexion of the mucous tunic, while, the firm union of the intestine itself, with the surrounding parts, the longitudinal direction of its strongest and most numerous fibres, together with the action of the levatores ani muscles, offer much resistance to the descent of the entire gut.

Mr. Syme takes a view which differs, in some respects, from the preceding. When only the mucous membrane is prolapsed, he refers the disorder to the head of hæmorrhoids. When all the coats are involved, he terms it "prolapsus ani."

"In this restricted sense," he proceeds "prolapsus ani consists of a tumour generally round or oval, but sometimes cylindrical, varying in size from that of a small egg to that of the largest orange, exhibiting the slimy surface of a mucous membrane, and affording a copious secretion of very similar appearance to red currant jelly. It is obvious that the connexions of the lower part of the rectum must prevent it from descending, so as to present these appearances, which can be accounted for only by supposing that the higher part of the gut becomes invaginated in the portion below it, so as to project beyond the anus. In short, the derangement will be the same as that which is named Intussusception, with this difference, that in the latter case the invagination occurs higher up the intestine, beyond the reach of sight and touch. It has been maintained by some that the lower part of the rectum alone was concerned in the formation of prolapsus, the protrusion of this apparently fixed portion being accounted for by the relaxation of its coats. But this explanation does not agree with the anatomical structure, the phenomena observed during reduction of the protruded bowel within the sphincter, or the appearances which have presented themselves in cases that terminated fatally." 90.

Dr. Bushe remarks that the amount of intestine displaced varies from a fold of the mucous membrane to several inches of the bowel itself. When the mucous membrane, he continues, is alone prolapsed in the child, it assumes the appearance of a small pyramidal, red, and coiled tumor; while in the adult it is less red, and generally takes the form, either of two lateral flaps, or of a circular fold. In some of these cases, the portion of membrane thus protruded comes from the pouch of the rectum, while that within the sphincters remains *in situ*. When this is the case, we can pass the extremity of the little finger between that portion of the membrane which adheres to the internal sphincter, and that which is protruded. This form of disease may be accounted for, by the comparatively greater extent of the mucous membrane of the pouch, and its looser adhesion to the muscular coat, than that which lies within the internal sphincter. It will be observed that Mr. Syme considers prolapsus of the rectum as identical with intus-susception. He does not state very clearly what part of the bowel is invaginated. But Dr. Bushe will be seen to deny the analogy. In protrusion of the rectum, he says, we are not able to insert a probe or finger higher than the border of the internal sphincter, in consequence of the doubling down of the mucous membrane, while in intus-susception no resistance is offered to the ascent of either one or the other. It is obvious that all hangs on the point *from which* the intus-susception commences. If it is low, we reach it—if high, we do not.

The consequences of the protrusion are such as might be expected. When it is allowed to remain down, says Dr. Bushe, it becomes engorged with blood from the pressure which the sphincter exercises on the veins, as manifested by its increase in size and livid colour. If it be not soon reduced, inflammation sets in, and is attended not only with great local pain, but fever, and in some rare instances, death ensues, in consequence of extensive peritoneal inflammation. In some other, and yet more rare cases, the protruding portion sloughs off, and a cure follows.

When the descent of the bowel is often repeated, the mucous membrane becomes indurated, loses its villous surface, and in some instances even ulcerates. This is more likely to be the case, when the sphincter has become relaxed from the repeated dilatation it has suffered, and there is a constant nisus causing the bowel to contract, and force out the mucous membrane. Such cases are generally hæmorrhoidal, and the persons so afflicted are miserable, as by no artificial means are they able to keep the membrane reduced for any length of time. Indeed, they can scarcely assume the erect position, cough, sneeze, or laugh, without suffering from its descent.

The symptoms vary with the amount of the protrusion, and with the vigour of the system; they are usually more urgent in young persons than in old.

B. *Causes.* Children and persons advanced in life are the two classes most prone to the complaint. Children are more prone to this disease, than adults, because the intestine is less curved; the sacrum is more perpendicular; the coccyx is not yet ossified, and is moveable on the sacrum; the connexions of the rectum are less extensive, in consequence of the imperfect development of the prostate, urethra, and vesiculæ seminales; the abdominal viscera are more voluminous; and finally, the mobility of the intestines is greater. In aged persons prolapsus is frequent, on account of the general relaxation of the tissues, and of the imperfect tone of the sphincter. Any thing which occasions great straining at stool may give rise to it. Thus constipation, hæmorrhoidal tumors, colitis, painter's colic, ascarides, severe cathartics, prolapsus uteri, parturition, stricture of the urethra, enlargement of the prostate gland, stone in the bladder, violent coughing, sneezing, &c., may be considered as so many occasional causes of this affection.

C. *Treatment.* It would be well if this were always as fortunate as could be wished. It admits of being naturally divided into—the reduction of the prolapsed bowel—and, the retention of it, when reduced.

*a. Reduction.* Dr. Bushe's directions on this head are more circumstantial than Mr. Syme's. We, therefore, extract them.

"In the treatment of prolapsus of the rectum, our first great object is to replace the protruded portion of the bowel. If recent, we may proceed to its reduction at once; but if it be engorged with blood or inflamed, leeches should first be applied to the surrounding parts, and the tumor itself fomented with a warm decoction of poppy heads. The patient being placed on his side in a recumbent position, and the buttocks separated by an assistant, the surgeon having oiled his fingers, endeavours, by a slow and steady compression, to diminish the size of the tumour, and then to push it within the internal sphincter. He should be careful not to introduce his finger within the anus, unless it be absolutely necessary; else, in withdrawing it, especially in children, a portion of the bowel will be again prolapsed. Some authors, fearing this difficulty, have with more ingenuity than practical skill, recommended the reduction to be accomplished with a distended gut, from which the air should be let out when the protrusion is replaced, and then withdrawn. Sir C. Bell recommends the point of the finger to be armed with a cone of paper, wetted at the point, and oiled on the outside; this, he says, will easily slip out, without bringing down the bowel. I can scarcely think that this will ever be necessary; but at all events, it is a very superior expedient to the distended gut. Should the sphincter be so contracted, as to prevent the return of the prolapsed portion of the intestine, the fissure knife ought to be cautiously introduced, and this muscle completely divided." 207.

The only hint which Mr. Syme offers, is—that the patient be desired not to hold his breath during the attempt at reduction. But as children are most commonly the subjects of the disorder, probably such an injunction will be frequently useless.

b. *Prevention of displacement.* The means of effecting this must vary, of course, with the nature of the case and of the causes. The irritation, whatever it may be, should be removed. If it occurs, says Dr. Bushe, in infants who have been too long nursed, they must be weaned,—if in children badly nourished, they ought to be well fed,—if in delicate and relaxed persons, bark, iron, cold bathing, a substantial diet, a bracing atmosphere, and regular exercise will be necessary,—if from injury or disease of the spinal marrow, this organ should be treated according to the character of the affection,—if from division of the sphincter, the introduction or extraction of foreign bodies the repeated expulsion of indurated feces, or the distention of the bowel, advantage may be derived from the injection of the infusion of galls or catechu, of a solution of alum or acetate of zinc, &c. If a stone in the bladder is the source of irritation, it should be cut out. If dentition has given birth to it, the gum should be scarified, and the ordinary remedies employed. Costiveness should be prevented by gentle laxatives; all drastic purgatives should be avoided.

Mr. Syme offers some judicious cautions with regard to the habit, on the part of patients, particularly children, of straining at stool. This should be guarded against as much as possible. With this view, the bowels ought not to be evacuated in the sitting posture usually assumed by children in doing so, as it renders the pressure of the diaphragm most direct upon the contents of the pelvis: and the patient should sit upon a chair so high as to prevent his feet from reaching the ground, which will keep the trunk erect, and moderate the force of the expulsive efforts. Care also should be taken to prevent him from sitting too long or too frequently at stool.

Dr. Bushe presents us with such a host of means of routing ascarides, that we are tempted to extract the catalogue. It is very hard if any are suffered to exist amid such a multiplicity of remedies.

"If ascarides nestle in the rectum, and by the irritation they create, give rise to prolapsus of the mucous membrane, or a portion of the intestine itself, we ought to inject some of the following preparations, viz: a decoction of wormwood and rue,—a decoction of chamomile flowers with castor oil and salt,—aloes suspended in milk, or rubbed up with oil,—lime-water and oil,—camphor, turpentine, or the essential oils, suspended in water with the white of egg,—sulphuret of potass in water, tincture of the muriate of iron in water,—or, tobacco smoke. Calomel and jalap, or aloetic purges, should be administered at the same time. In some few cases, they may be so numerous as to require extraction." 208.

When the protrusion depends on defective resistance of the sphincter, the great object is to augment that resistance or to find a substitute for it. Constipation of the bowels, says Mr. Syme, which necessarily leads to long-continued and laborious efforts at expulsion, should be carefully prevented by regulation of the diet and regimen; by the use of appropriate laxatives; and by the injection of tepid water into the rectum, which not only is a powerful assistant of medicines given by the mouth, but often proves sufficient to supersede their employment altogether. The enema syringe of Read, more convenient, perhaps, in its original form than any of the subsequent modifications which it has undergone, renders the administration of this means so easy and simple, that no difficulty need be experienced in its habitual use. Every patient who suffers from constipation of the bowels, and more especially all those who have any tendency to prolapsus, should be provided with the apparatus.

When the weakness of the sphincter depends upon a paralytic state of the muscle, little can be done for its remedy, and a bandage must be worn to support the rectum.

But if the sphincter is not actually paralytic, but only relaxed, an operation may be serviceable, perhaps may effect a cure. Dr. Bushe thus points out the circumstances which call for, and which modify the operation.

"Should the means recommended," he says, "fail, we must proceed to a surgical operation, the nature of which will depend upon the character of the disease. Thus, when there is a considerable prolapsus, and the sphincter does not appear much relaxed, one, two, three, or four portions of the mucous membrane, according to the extent of the disease, each about the size of a shilling, ought to be pinched up with a forceps, on opposite sides of the bowel, at different altitudes from the sphincter, then tied after the manner of hæmorrhoidal tumours, and finally snipped off outside the ligatures, with a curved scissors. The prolapsus should then be reduced, the patient put to bed, and a dose of morphine exhibited. On the second day, the bowels must be opened with an enema consisting of oil and gruel, and this should be afterwards repeated, as often as may be necessary. The ligatures are generally cast off within ten days, after which, the healing process goes on rapidly, and so firm does the adhesion become, that the prolapsus gradually disappears.

If the sphincter be relaxed, and the folds of fine skin at the anus elongated, an operation nearly similar to that first performed by the late Mr. Hey of Leeds in England, ought to be preferred. It consists in excising some of the mucous membrane and fine skin of the verge of the anus, so as to give rise to more firm adhesion of the remaining mucous membrane and skin, to the subjaeent parts. To perform this operation, the patient should lean over the back of a chair, or else be placed in the same position as for lithotomy; then, the operator removes as many folds of the fine skin and mucous membrane, as he may think necessary, by means of a slender dressing forceps, and curved scissors. The subsequent treatment, should be similar to that recommended after the operation by ligature. In a fortnight or three weeks, the wounds will have healed, and the adhesion become so firm, as to enable the anus to support the bowel.

I have repeatedly performed both of these operations; but the latter, with the most success.

Should lateral flaps of the mucous membrane hang down, in the adult, as described at page 204, they ought to be seized with a forceps, and cut off with a knife or curved scissors; or, they may be removed with ligatures, after the manner of hæmorrhoidal tumours. These cases are exceedingly common, in proof of which, I have operated on four in eleven weeks." 211.

Mr. Syme's recommendations coincide very nearly with those of Dr. Bushe.

The only additions which the former makes to the observations of the latter are the following:—The folds of skin should be held tense by a hook or forceps, and be removed from the distance of about an inch and a-half quite up to the mucous membrane, a small part of which should be included in the incision. It is not necessary to remove more than four or five of the folds. Mr. Howship has recommended the ligature, instead of the knife or scissors, for this purpose, on the ground that it excites a more salutary degree of irritation. But the pain and delay attending its use would more than counter-balance this alleged advantage, which may be compensated for by the freedom of excision.

Dr. Bushe adds a word or two on the management of the case, when the mucous membrane becomes indurated and partially ulcerated. It should be, under those circumstances, excised.

*Case.* Mrs. A. had been affected with protrusion of the mucous membrane for seventeen years. When she consulted Dr. Bushe, she was worn down by pain, purulent discharge, and confinement, for the moment she stood erect, the protrusion occurred. She had tried all possible local remedies, as leeches, fomentations, anodyne and astringent lotions and ointments, at the same time that she took internally an endless catalogue of drugs, and gave sea bathing and sulphureous waters a full trial. On examination, Dr. B. found that the protruded membrane consisted of a circular fold, which was very hard, and in many points ulcerated. When reduced, it felt like a thick welt, or cartilaginous ring, and was immediately prolapsed by the erect position, coughing or sneezing. Dr. B. removed the diseased membrane, and a perfect cure ensued.

When the patient will not submit to the operation suited to his case, his condition may be palliated, by wearing the truss recommended by the late Mr. Gooch.*

We have now presented a complete, and, we hope, an useful practical account of two frequent, troublesome, remediable, and, therefore, important diseases—hæmorrhoids, and prolapsus of the rectum. We are sure that much of what we have extracted from the works of both the authors before us, but more especially from that of Dr. Bushe, will prove of value to such of our readers as are actually engaged in practice. We shall return to the diseases of the rectum in our next number, and we hope to present a complete series of articles upon the subject.

---

* The Chirurgical Works of Benjamin Gooch. London, MDCCXCII. vol. ii. p. 150.

